# Supplementary material for: Stretchable piezoelectric biocrystal thin films
Source: Nat Commun. 2023 Oct 17;14:6562. doi: 10.1038/s41467-023-42184-8 (PMC10582159; doi:10.1038/s41467-023-42184-8)
Supplement: Supplementary file 1 — Supplementary Information [file 41467_2023_42184_MOESM1_ESM.pdf]

Stretchable Piezoelectric Biocrystal Thin Films

Jun Li,[1] Corey Carlos,[1] Hao Zhou,[2] Jiajie Sui,[1] Yikai Wang,[1] Zulmari Silva-Pedraza,[1,3] Fan Yang,[4] Yutao Dong,[1] Ziyi Zhang,[1] Timothy A. Hacker,[5] Bo Liu,[3] Yanchao Mao,[2,*] Xudong Wang[1,*]

[1] Department of Materials Science and Engineering, University of Wisconsin-Madison, Madison, WI 53706, USA

[2] Key Laboratory of Materials Physics, Ministry of Education, School of Physics and Microelectronics, Zhengzhou University, Zhengzhou, 450001, China

[3] Department of Surgery, School of Medicine and Public Health, University of Wisconsin-Madison, Madison, WI 53705, USA

[4] Department of Orthopaedics, Shanghai Key Laboratory for Prevention and Treatment of Bone and Joint Diseases, Shanghai Institute of Traumatology and Orthopaedics, Ruijin Hospital, Shanghai Jiao Tong University School of Medicine, Shanghai, Shanghai, 200025, China

[5] Cardiovascular Research Center, University of Wisconsin–Madison, Madison, WI 53705, USA

E-mail: XW: xudong.wang@wisc.edu; YM: ymao@zzu.edu.cn

This file includes:

Supplementary Methods
Supplementary Text
Figs. S1 to S25
Table S1

Other Supplementary Materials for this manuscript include the following:

Supplementary Movies S1 to S3

Supplementary Methods

Calculation of piezoelectric charge coefficients (d_{eff}):

The effective piezoelectric charge coefficients (d_{eff}) of MF network were estimated from measured charge outputs Q and applied mechanical force F, following the process shown below:

$$d_{eff} = \frac{Q}{F} = \frac{\int Idt}{F}$$

Determination of piezoelectric charge coefficients (d_{eff}):

The definition of piezoelectric coefficient follows the standard definition of piezoelectricity:

$$P_i = d_{ij}\sigma_j \; (i = 1, 2, 3; \; j = 1, 2, ..., 6)$$

where P is the polarization, d is the piezoelectric coefficient and σ is the stress. As illustrated in Figure R6, the out-of-plane direction is "3" direction of DL alanine/PDMS film, where the in-plane longitudinal direction (growth direction) and transverse direction are "1" direction and "2" direction, respectively. Since the parallel electrodes were placed to collect the longitudinal polarization built on the "1" direction of network and the tapping force is applied along its "3" direction, the effective piezoelectric coefficient we measured is the d_{13}^{eff} of the entire DL alanine network film.

Calculation of efficiency of piezoelectric network:

Here, in our work, the energy conversion efficiency η of DL-alanine network/PDMS film can be estimated by comparing the input energy and output energy:

$$\eta = \frac{E_{output}}{E_{input}}$$

The energy input by tapping force (3 N) into the DL-alanine/PDMS film (Figure 3d) is equal to the energy built by strain in the network/PDMS film and can be calculated using the following equation:

$$E_{input} = \frac{1}{2} SE\varepsilon^2$$

where S is the effective volume of the film (10 um×1 cm×1 cm), E is the Young's modulus (3.8 MPameasured in the manuscript), and ε is the average strain (~0.5 % under 3 N normal tapping force, estimated based on the film deformation). Meanwhile, the output energy of DL-alanine/PDMS thin film can be calculated by the formula:

$$E_{output} = \int_{t_1}^{t_2} \frac{V^2}{R} dt$$

R is the inner impedance of voltage meter (10 MΩ), V is the measured voltage. The E_{input} is obtained as ~48 nJ whereas E_{output} is 0.52 nJ. Therefore, the energy efficiency therefore is approximately 1.08%.

Calibration of PFM by standard $LiNbO_3$ sample:

A periodically poled lithium niobate (PPLN) specimen from Bruker was utilized as a reference sample for PFM calibration. This PPLN has a known effective piezoelectric coefficient d_{33} = 7.5 pm/V. First, we measured the vertical piezoelectric response of the calibration sample (Figure R3a and b). We then estimated the slope k_{PPLN} of the linear fitting of the piezoresponse amplitude versus the applied voltage (Figure R3c). Afterward, the calibration factor α can be obtained as:

$$\alpha = k_{PPLN} / d_{33-reference}$$

in which d_{33}-reference is the known piezoelectric coefficient of the reference sample. Therefore, the d_{33} of experimental sample is given by the consideration of calibration factor α:

$$d_{33} = \frac{d_{measured}}{\alpha \times V_{AC}}$$

where $d_{measured}$ is the and V_{AC} is the alternating driving voltage. As shown in Figure S13, our calculated piezocoefficient of PPLN (k_{PPLN} = 8.2 pm/V) is very close to the reference value ($d_{33-reference}$ = 7.5 pm/V). As a result, we only have a calibration factor α = 8.2/7.5 ≈ 1.1. This calibration factor has been included in the calculation of effective d_{33} of DL alanine MFs.

Supplementary Text

In the process of second-harmonic generation, the nonlinear polarization created in piezoelectric crystals can be described by the following equation:

$$P_i(2\omega) = 2\varepsilon_0 \sum_{jk} d_{ijk} E_j(\omega) E_k(\omega)$$

where ε_0 is the permittivity of free space, d is the second-order non-linear coefficient, and E is the electric field. The symmetry requirements for both SHG and piezoelectricity are identical, as both are described mathematically by the third rank tensor d_{ijk}.

The above equation can be written in matrix notation as below:

$$\begin{bmatrix} P_x(2\omega) \\ P_y(2\omega) \\ P_z(2\omega) \end{bmatrix} = \varepsilon_0 \begin{bmatrix} d_{11} & d_{12} & d_{13} & d_{14} & d_{15} & d_{16} \\ d_{21} & d_{22} & d_{23} & d_{24} & d_{25} & d_{26} \\ d_{31} & d_{32} & d_{33} & d_{34} & d_{35} & d_{36} \end{bmatrix} \begin{bmatrix} E_x(\omega)^2 \\ E_y(\omega)^2 \\ E_z(\omega)^2 \\ 2E_y(\omega)E_z(\omega) \\ 2E_x(\omega)E_z(\omega) \\ 2E_x(\omega)E_y(\omega) \end{bmatrix}$$

Given that DL-alanine crystal has mm2 point group, its second-order non-linear coefficient matrix is:

$$\begin{bmatrix} 0 & 0 & 0 & 0 & d_{15} & 0 \\ 0 & 0 & 0 & d_{24} & 0 & 0 \\ d_{31} & d_{32} & d_{33} & 0 & 0 & 0 \end{bmatrix}$$

The intensity of the outgoing SHG light is:

$$I^{(2\omega)} \sim (I_0)^2 F(\Theta, \Phi, 2\omega)^2 f(\vartheta, \varphi, \omega)^2 \begin{bmatrix} 0 & 0 & 0 & 0 & d_{15} & 0 \\ 0 & 0 & 0 & d_{24} & 0 & 0 \\ d_{31} & d_{32} & d_{33} & 0 & 0 & 0 \end{bmatrix}$$

where I is the intensity of the incident light, F() and f() describe Fresnel and geometrical factors for the incident and reflected light, respectively, Θ and θ are angles of reflection light and incidence light, respectively, and Φ and φ are output polarization and input polarization angle, respectively. When gradually adjusting the polarization direction of the light by a step of 10 degree, the intensity of SHG signal would incorporate the symmetry of DL-alanine crystals according to the Neumann's principle, which is "any type of symmetry which is exhibited by the crystal is possessed by every physical property of the crystal." [1,2].

S2: Supplementary Figures

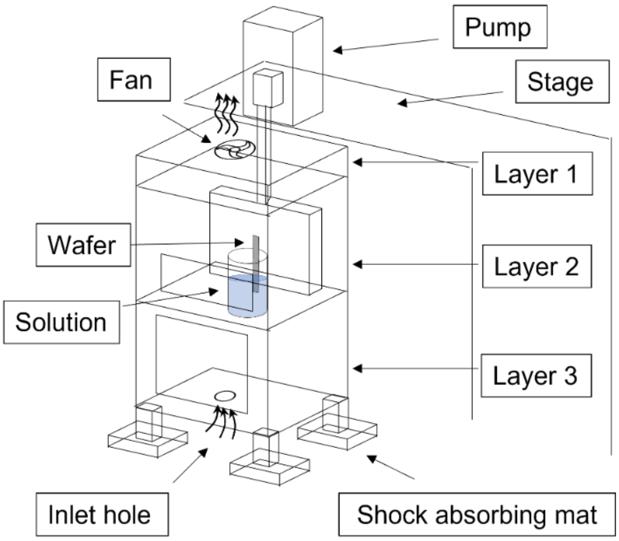

Figure S1: Schematics of the DL-alanine MF network growth system. This system mainly consists of a syringe pump, three-layer growth chamber, and an air flow system. The syringe pump controls the pulling out speed of wafer substrate from the biphasic solution. Both layer 1 and layer 3 in the chamber are filled with desiccants to adjust and stabilize the humidity in layer 2. Layer 2 is the space with constant temperature (23 °C) and humidity (15%) where MF network growth happens. Air flow was regulated by tuning the power of fans so that the convention flow and evaporation speed are set.

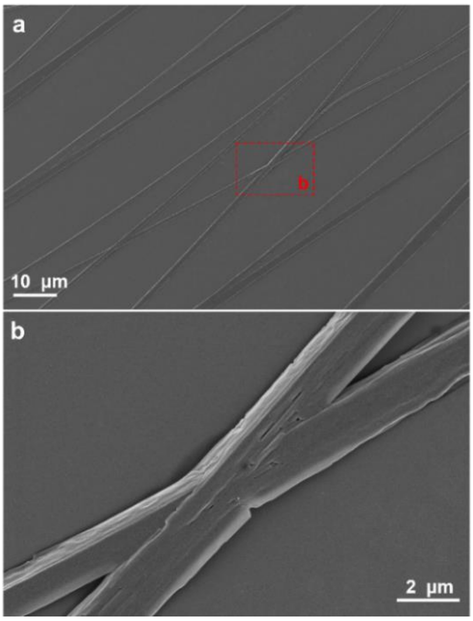

Figure S2. Consecutive Merging and Bifurcation Region in DL-alanine Network. a. Low-magnification SEM image of the region. **b.** High-magnification zoomed-in SEM image of the red boxed area in A showing a X-shaped junction. Representative image from experiments (n =10).

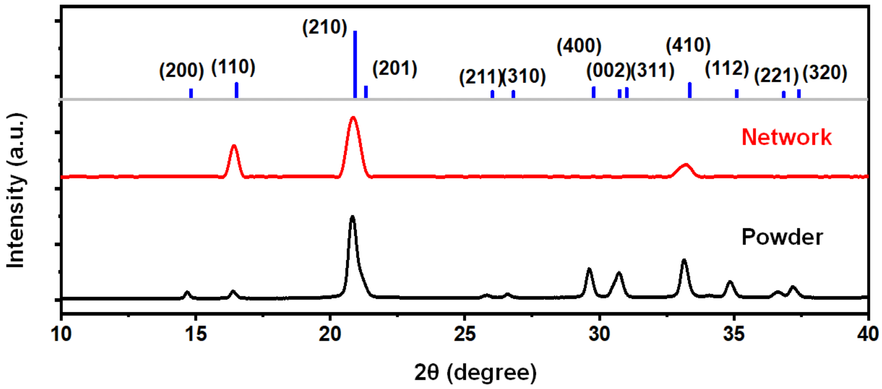

Figure S3: XRD of DL-alanine MF network and DL-alanine powders. The standard XRD spectra (PDF#21-1569) of orthorhombic DL-alanine (Pna2_1) are given on top.

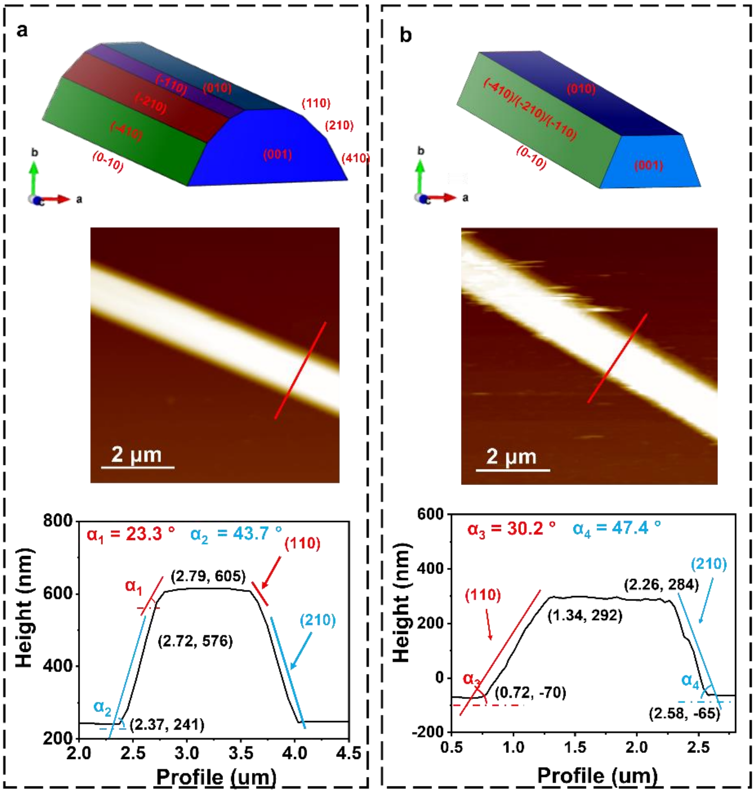

Figure S4. Crystal Orientation and Crystal Facets of DL-alanine MF. a. Schematic image and AFM image of a trapezoid belt shaped MF with smooth sides including all (110), (210) and (410) facets. The bottom figure is the height profile of the MF following the red dashed line in the AFM image. **b.** Schematic image and AFM image of a trapezoid belt shaped MF with each leg side consisting of only one facet ((110) or (210) or (410)). According to the crystal information of DL-alanine (PDF#21-1569), the theoretical angles between (410) and (010), (210) and (010), (110) and (010) are 63.51 °, 45.09 °, and 26.64 °, respectively. The real angles between different facets can be calculated based on coordinates of turning points in AFM height profiles of single MFs.

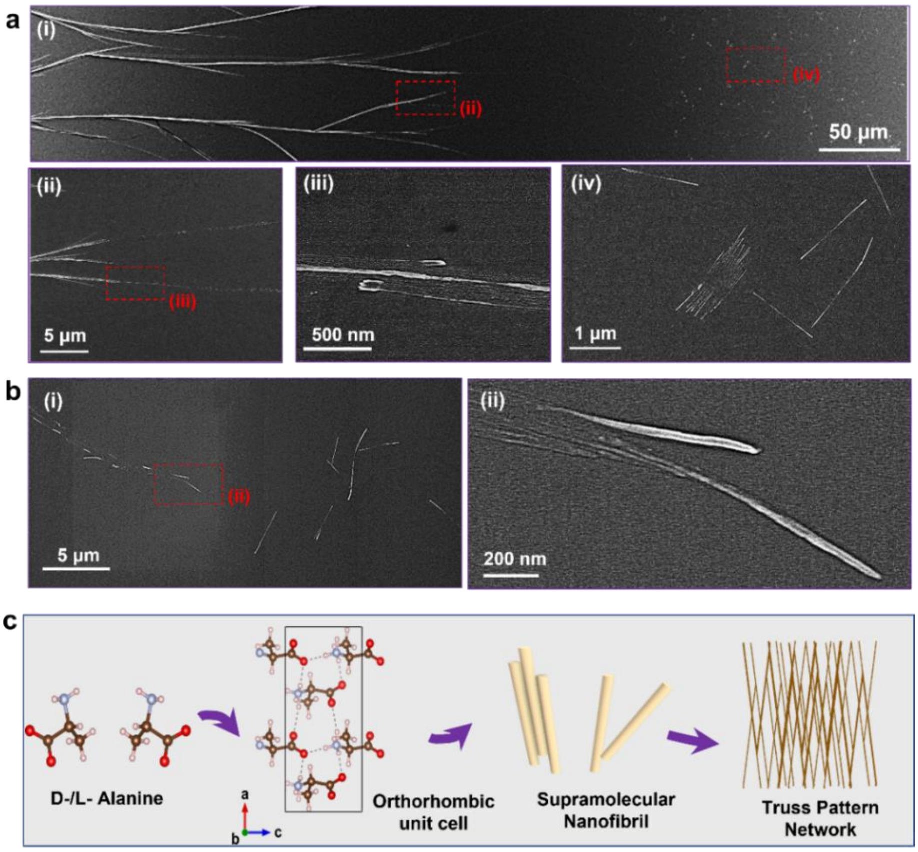

Figure S5: DL-alanine MF Network Self-Assembly. **a.** SEM images of the growth front of the MF network. Three distinctive regions are observed, namely, well-faceted interconnected MFs, less faceted fine MFs, and dispersed nanofibrils. **b.** SEM images of the tip region of a forming DL-alanine MF. The tip is formed by attaching the nanofibrils to the existing DL-alanine MF. **c.** The schematics of the formation process of the truss pattern network. D-alanine and L-alanine molecules are first packed into nanofibrils. These nanofibrils assembled into MFs that intersected with each other.

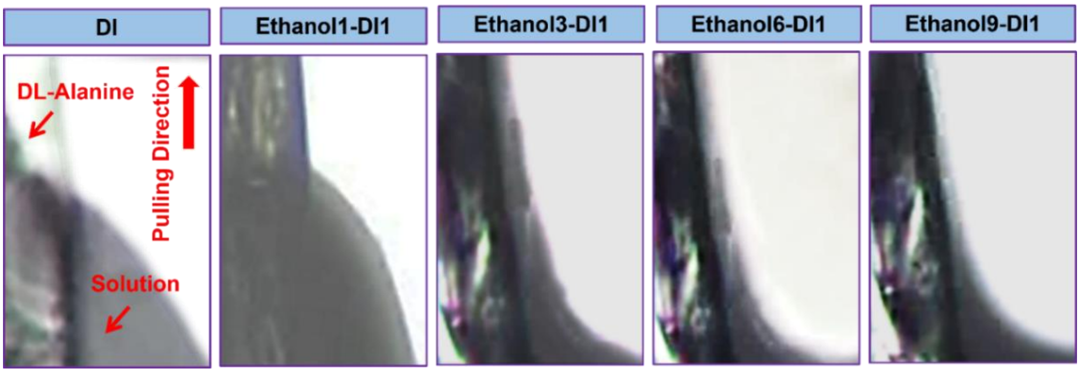

Figure S6: Contact angles of different solutions on DL-alanine micro-crystals by optical microscopes. The solutions are water and biphasic solutions consisting of water and ethanol with varying compositions. The difference in contact angles is associated with different surface tension that deflects of attachment of nanofibrils. The pulling direction, DL-alanine MF and solution are indicated by red arrows.

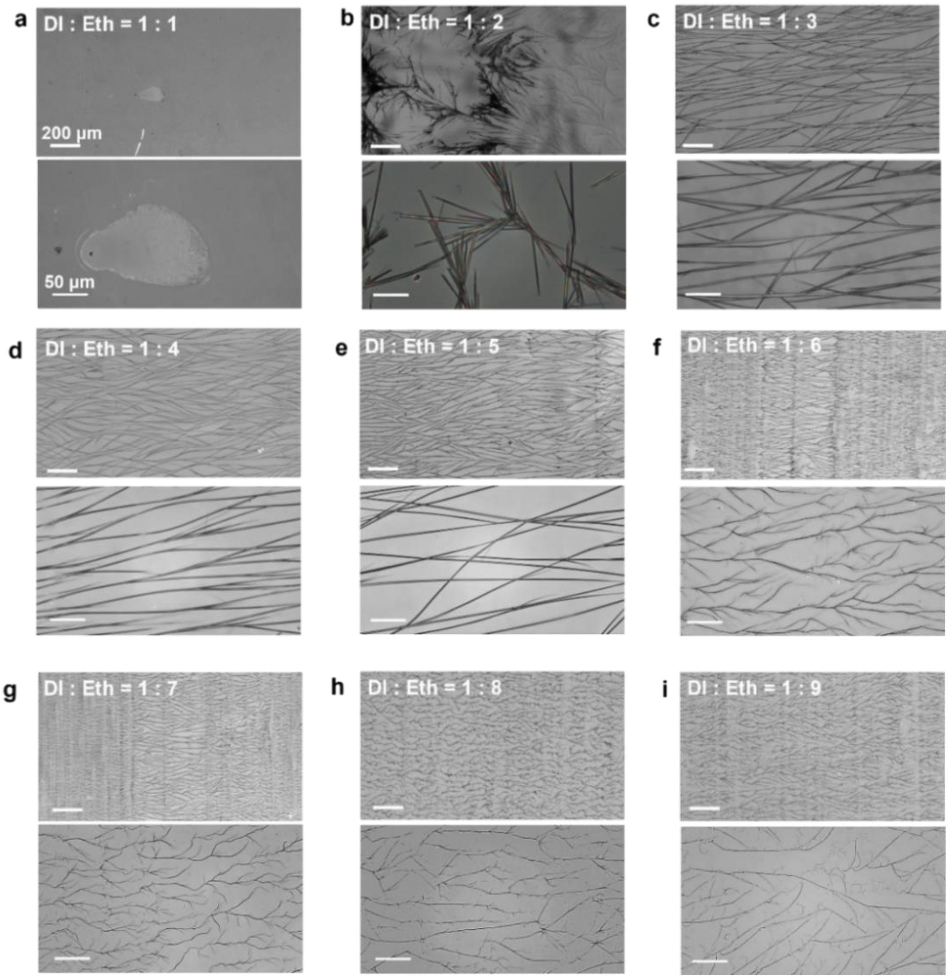

Figure S7: DL-alanine Biocrystals by Different Solutions. a to **i** are optical images of precipitated DL-alanine biocrystals from solutions with water to ethanol volume ratio varying from 1:1 to 1:9. Representative image from experiments (n = 18) .

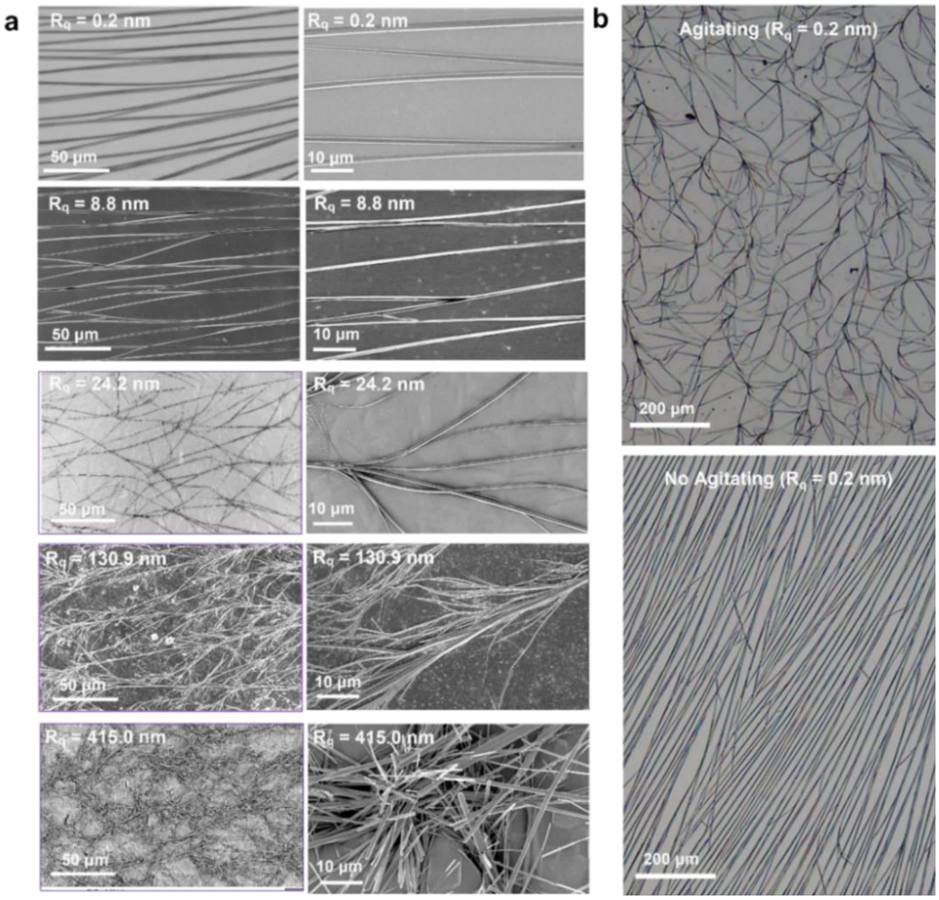

Figure S8: DL-alanine MF Bifurcation under Instable Meniscus. a. SEM images of interconnected networks and branched MFs and on Si wafer substrates with different roughness. While bifurcation was found at very few intersection regions on smooth substrates (Rq = 0.2 nm). More MF could branch into multiple ones on rough substrates (Rq ≥ 8.8 nm). Higher branch density led to less interconnected microfiber network, as the growth of many branched fibers terminated shortly due to the competing for precursors. On extremely rough surface (Rq = 415.0 nm), no continuous truss patterned network was formed, instead, only short and heavily aggregated rods were observed. **b.** SEM images of DL-alanine networks growing on same-flatness substrates with/without agitating the biphasic solution

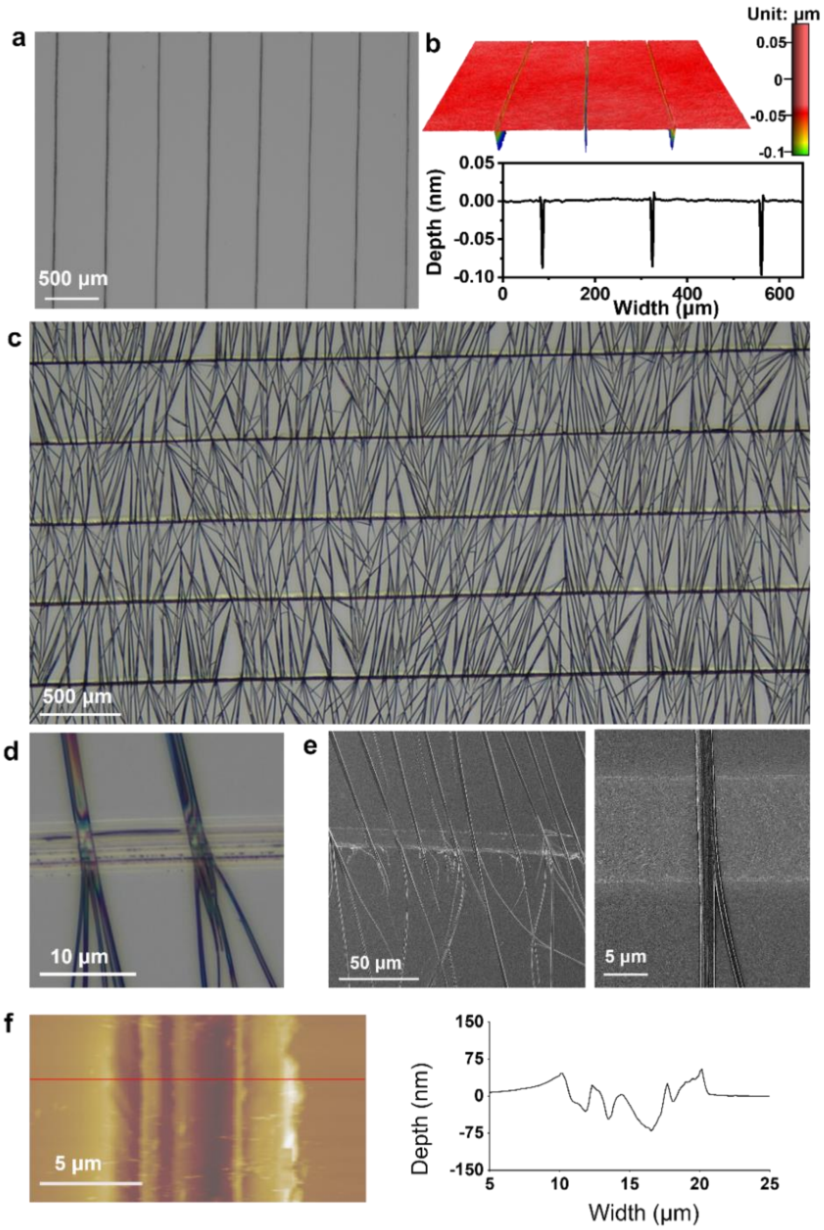

Figure S9. DL-alanine Network with Selective Branching and Intersection. a. Optical microscope image of patterned fine trenches on a Si substrate. The trenches were 500 µm apart from each other. **b.** Three-dimensional profile of the trench array measured by an optical interferometer. The bottom figure is the depth profile of the created trench. Each trench has a depth of ~80-90 nm and a width of ~10 µm **c.** Large-scale optical image of DL-alanine MF network grown on the Si substrate with trenches. The DL-alanine MFs selectively branched at the trenched area, suggesting a possibility of defining the truss mesh dimensions. **d.** Optical images showing the branching phenomenon occurred right after the MF crossing the trench. **e.** SEM images of DL-alanine MFs crossing over a trench and a single MF branching at the trench (right image). Fine but rough trenches were created on the flat wafer substrate (Rq = 0.2 nm). Branching was rarely observed before the DL-alanine micro-belts crossing over the trenches, whereas substantial bifurcations unambiguously appeared in the rough trench regions. **f.** Local AFM image of the trench and height profile of the trench as marked by the red line.

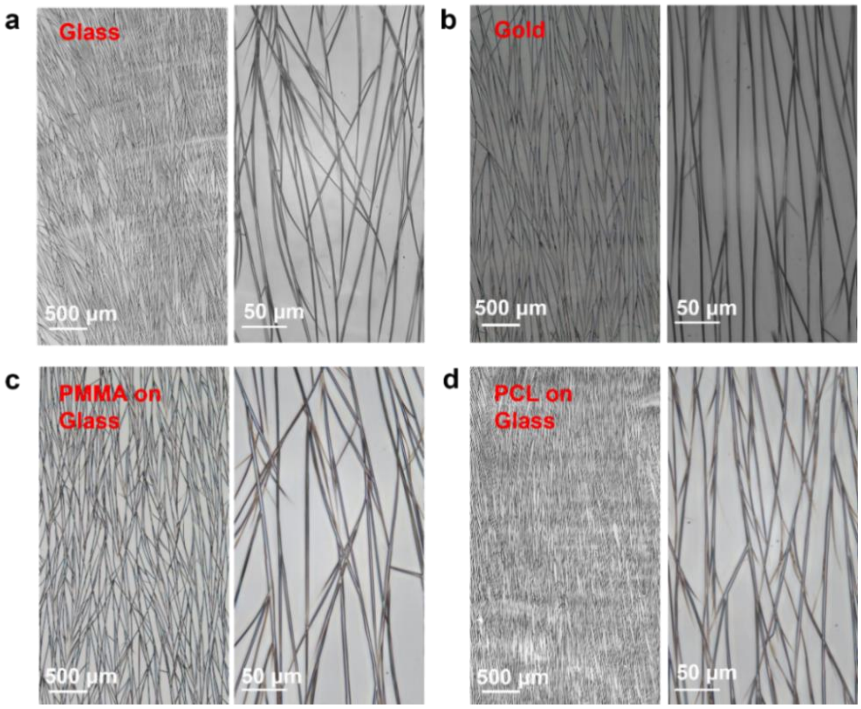

Figure S10. Optical Images of DL-alanine MF Networks on Versatile Substrates. This self-assembly strategy of DL-alanine network is fairly versatile and can be achieved on a large variety of hydrophilic substrates, including ceramics (e.g., glass **a.**), metals (e.g., gold **b.**), and polymers (e.g., poly(methyl methacrylate) (PMMA) **c.** and polycaprolactone (PCL) **d.**)

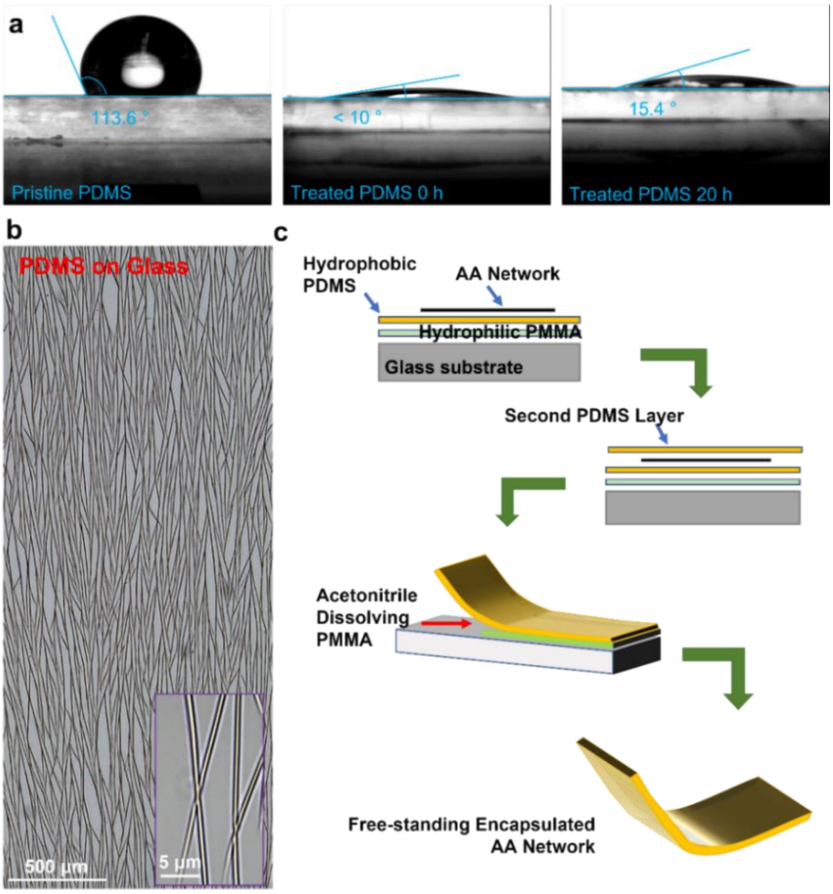

Figure S11: **DL-alanine MF Networks on PDMS Elastomer. a.** Contact angles of PDMS before and after oxygen plasma treatment. **b.** Optical microscope images of DL-alanine MF network grown on hydrophilic PDMS substrate **c.** Schematics of obtaining free-standing DL-alanine network/PDMS film by dissolving sacrificial PMMA layer.

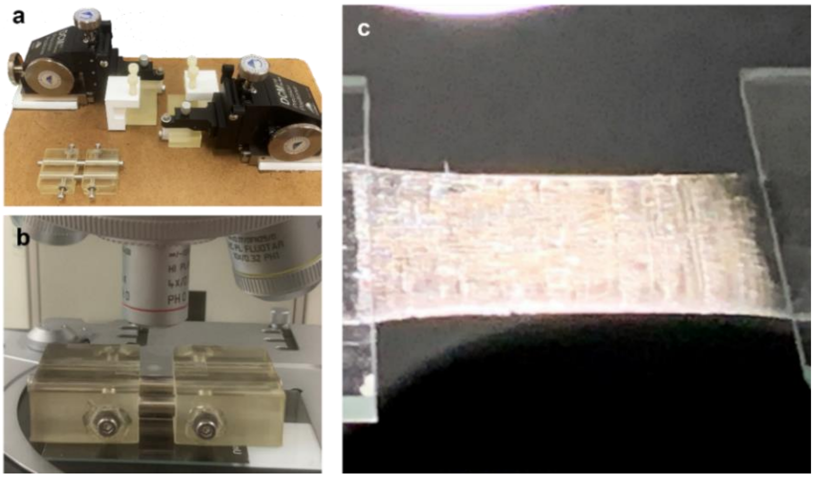

Figure S12: DL-alanine MF Networks Straining Test. Digital images of customized straining system for testing the MF network stretchability **a.**, optical microscope in-situ observing the straining process **b.**, and longitudinally strained MF network on PDMS substrate **c.**.

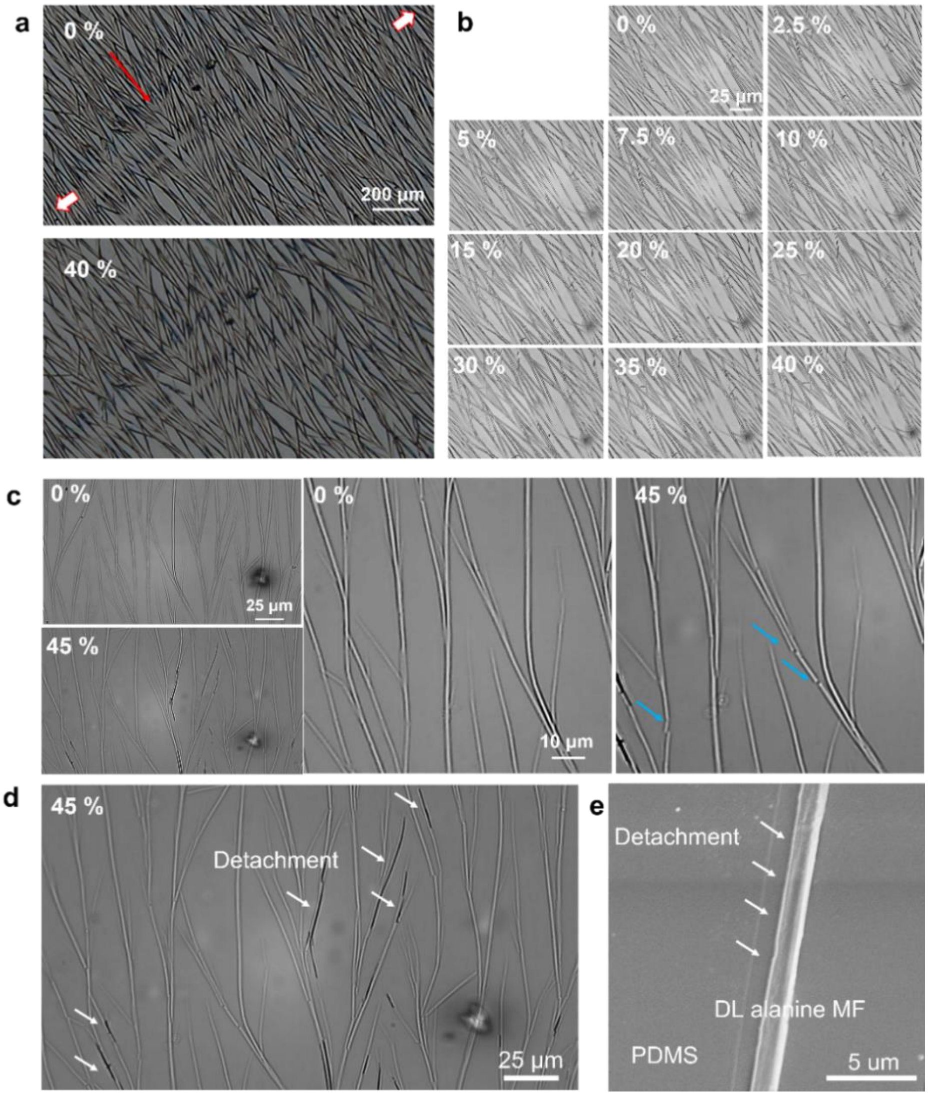

Figure S13: DL-alanine MF Networks under Transverse Tensile Strain. **a.** Large area optical microscope images of DL-alanine MF network under 0% and 40% tensile strain. The red arrow and white arrows indicate the growth direction and strain direction, respectively. **b.** High-magnification optical microscope images DL-alanine MF network under a series of transverse tensile strain ranging from 0% to 40%. **c**. Optical microscope images of DL-alanine MF network experiencing 45% transverse tensile strain. Broken MFs were observed (indicated by blue arrows) in network with 45% strains. **d.** Detachment of DL-alanine MFs from substrate after 45% transverse strains. **e.** SEM image of a single DL-alanine MF detached from substrate

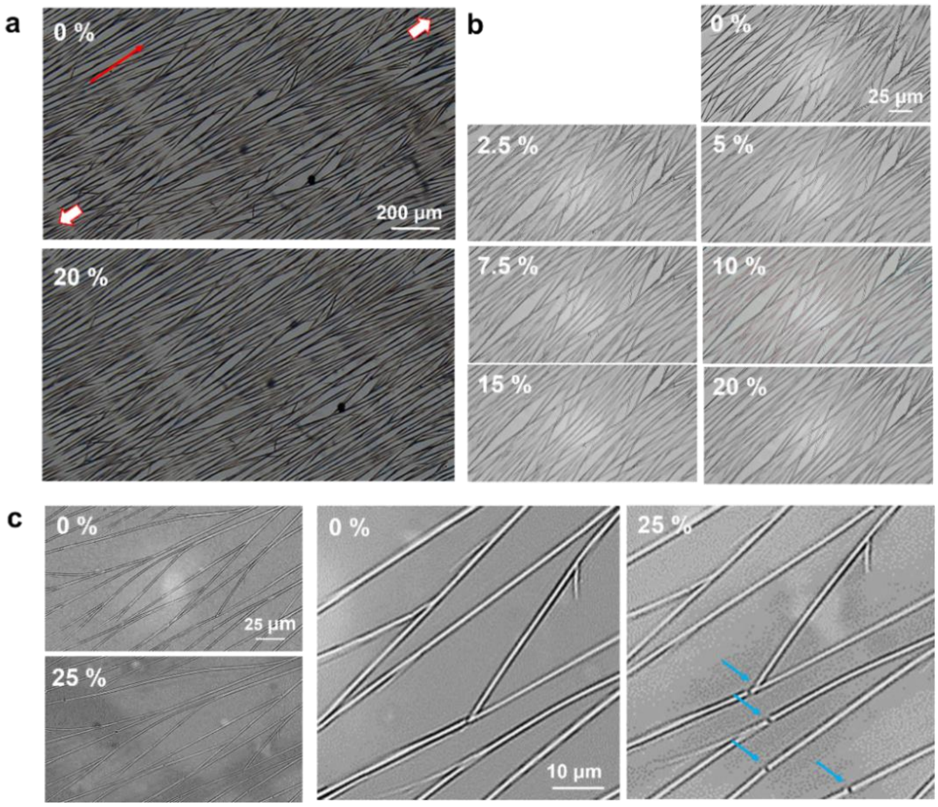

Figure S14. DL-alanine MF Networks under Longitudinal Tensile Strain. **a.** Large area optical microscope images of DL-alanine MF network under 0% and 20% tensile strain. The red arrow and white arrows indicate the growth direction and strain direction, respectively. **b.** High-magnification optical microscope images DL-alanine MF network under a series of transverse tensile strain ranging from 0% to 20%. **c.** Optical microscope images of DL-alanine MF network experiencing 25% longitudinal tensile strain. Broken MFs were observed (indicated by blue arrows) in network with 25% strains.

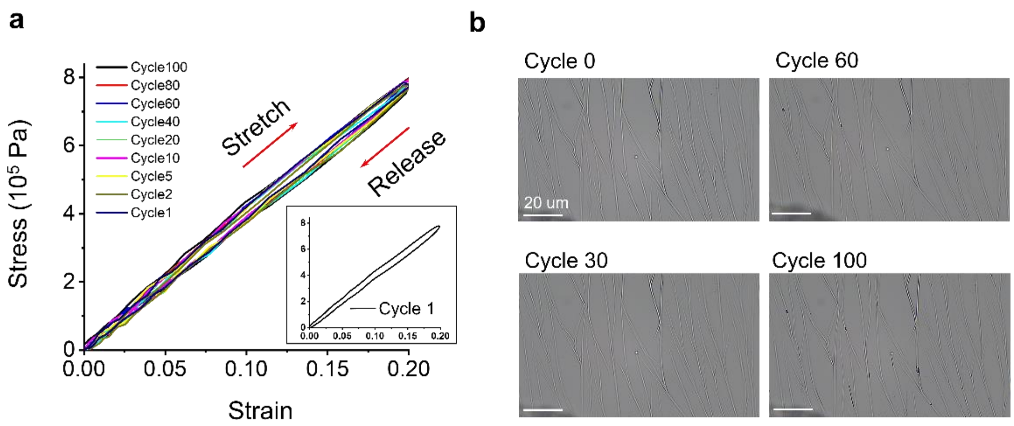

Figure S15. Fatigue test of DL-alanine network. a. Cyclic loading-unloading tensile straining of DL-alanine network on PDMS substate. **b.** Optical microscopes of DL-alanine network after cycles of straining. The few dark-contrast spots in cycle 60 and 100 images indicate possible small deviations from the original structure.

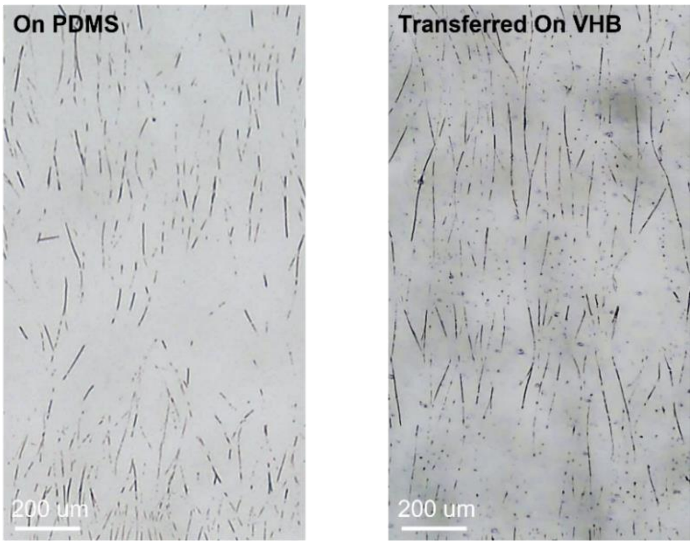

Figure S16. Transferring DL-alanine network from PDMS substrate to commercial VHB tape. The left image is the DL-alanine fragments left on PDMS after transferring. The right image is the transferred DL-alanine fragments on VHB tape.

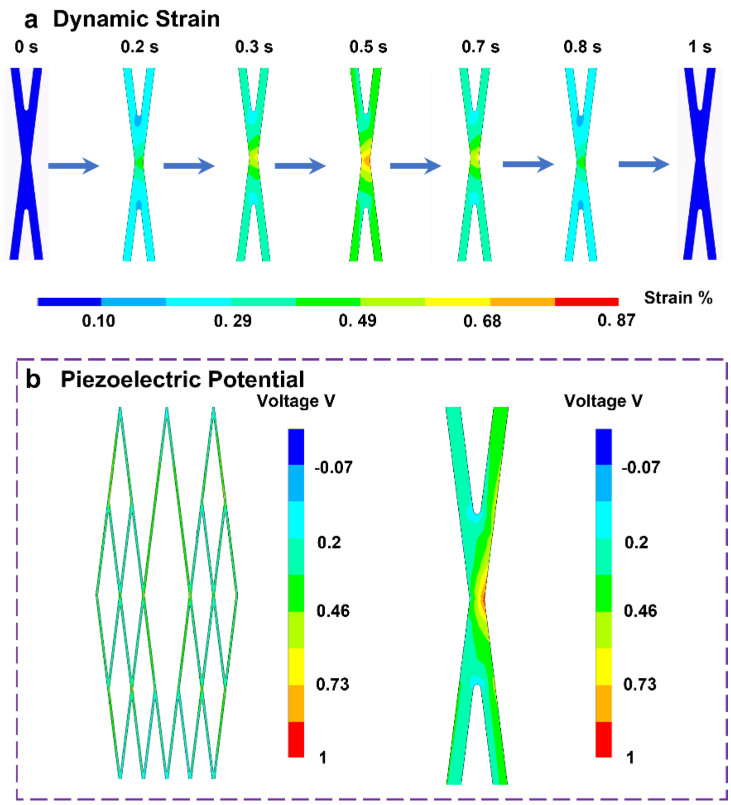

Figure S17. **Network Simulations. a.** Dynamic strain distribution of an "X" junction at different time frames with the network under unidirectional dynamic loading (deformation Y $\propto$ $\sin^2(\pi t)$). **b.** The piezoelectric potential distribution of one complete quadrilateral unit and an "X" junction under 20% longitudinal tensile strain.

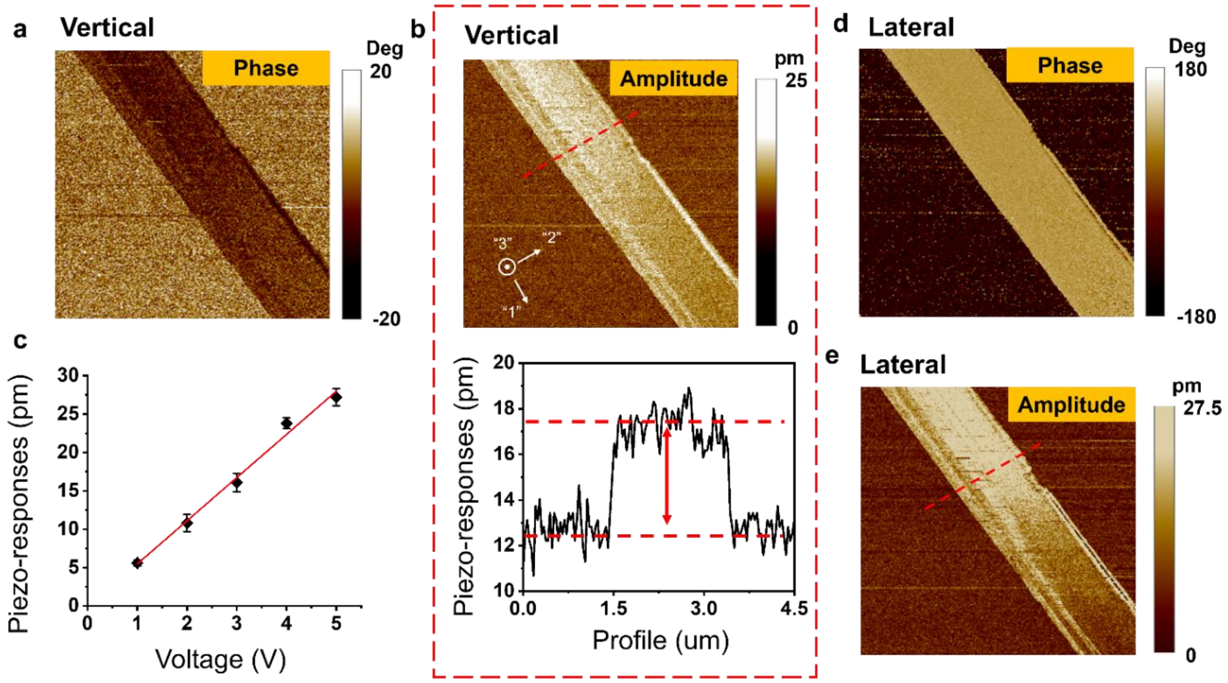

Figure S18. Piezoelectricity of single DL-alanine MF. a. Vertical PFM phase response of single DL-alanine MF. **b.** Vertical PFM amplitude response of single DL-alanine MF. Effective directions in relation to the DL alanine MF are defined by the white arrows. The out of plane direction is thus defined as effective "3" direction. The subtraction of substrate background contribution in piezoresponses is highlighted. **c.** PFM amplitude responses of single DL-alanine under different driving voltages. **d.** Lateral PFM phase response of single DL-alanine MF. **e.** Lateral PFM amplitude response of single DL-alanine MF. Uniformly strong phase responses in vertical direction indicated high out-of-plane polarizations whereas piezoelectric coefficients d_{33}^{eff} can be assessed by amplitude responses under different driving voltages. As lateral PFM test usually couple both shear and transverse responses due to the complex torsional deflection of the AFM tip, it is challenging to assess specific transverse piezo-coefficient (e.g., d_{31}) and shear coefficient (e.g., d_{15}).

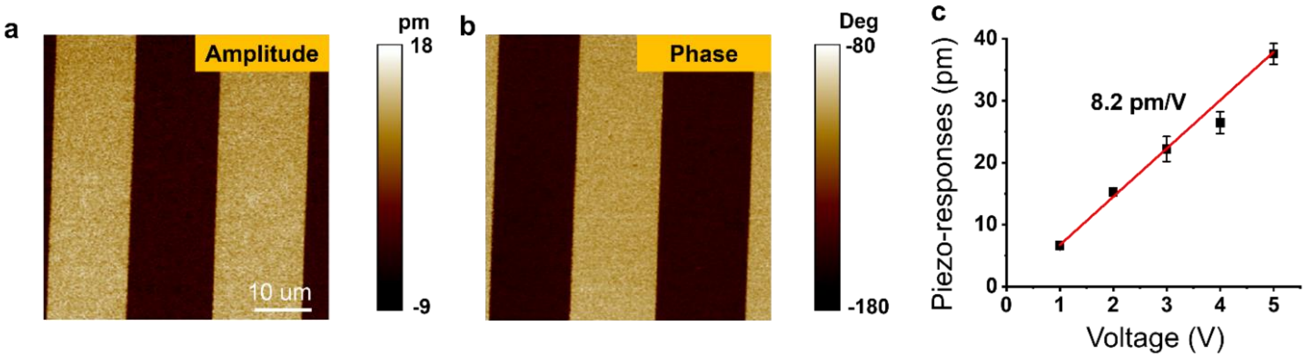

Figure S19. PFM calibration by standard periodically poled LiNbO3 (PPLN) sample. a. Vertical amplitude response of PPLN sample under 1 V driving voltage. **b.** Vertical phase response of PPLN sample under 1 V driving voltage. **c.** Linear fitting of the piezoresponse amplitude versus the applied voltage

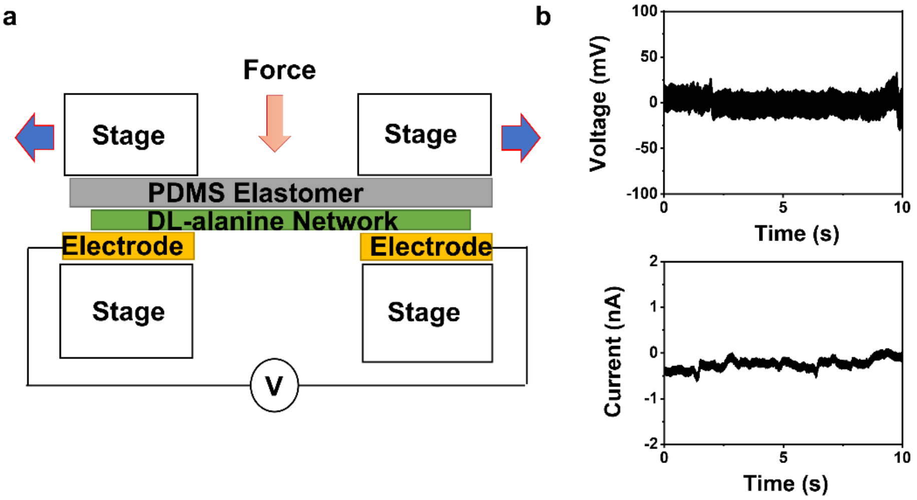

Figure S20. **a.** Schematic of experiment set-up for piezoelectric coefficient measurement. **b.** Voltage and current output of pure PDMS substrate without DL-alanine network.

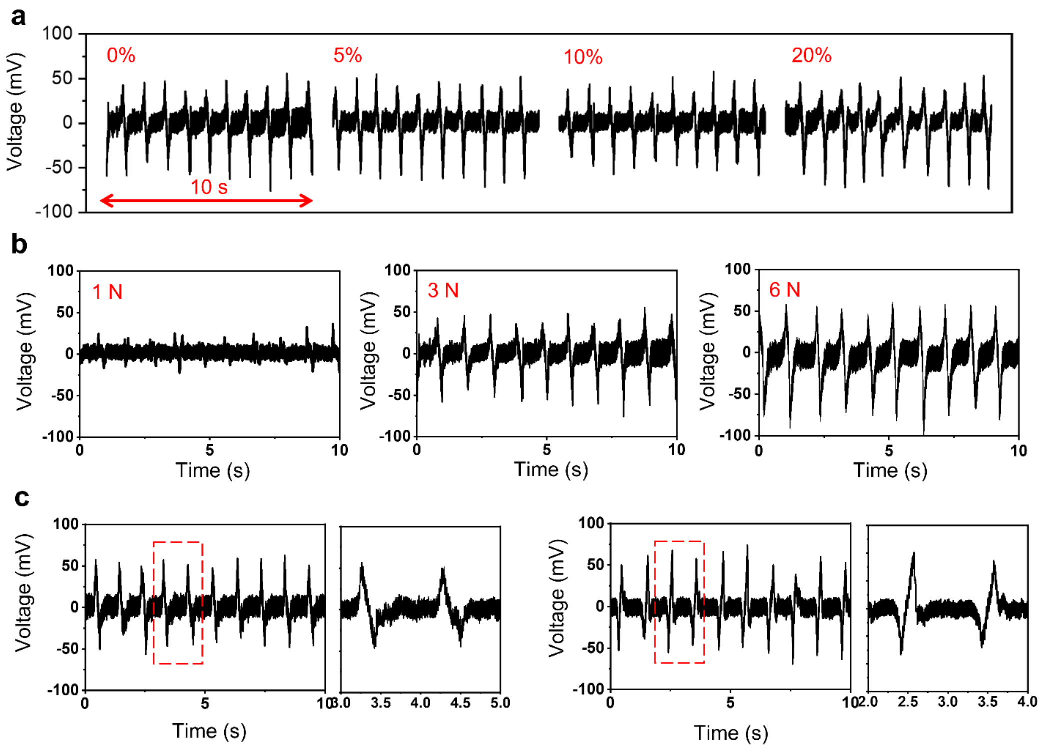

Figure S21. **Voltage outputs of MF network PDMS films. a.** Voltage output of DL-alanine piezoelectric network under a series of strains (0%, 5%, 10%, 20%). **b.** Voltage output of DL-alanine piezoelectric network under different forces (1-6 N). **c.** Voltage output of DL-alanine piezoelectric network under forward and reverse connections between electrodes and electrometer probes. Forward and reverse connections between electrodes and electrometer probes resulted in voltage outputs with opposite polarity, indicating real piezoelectric signals.

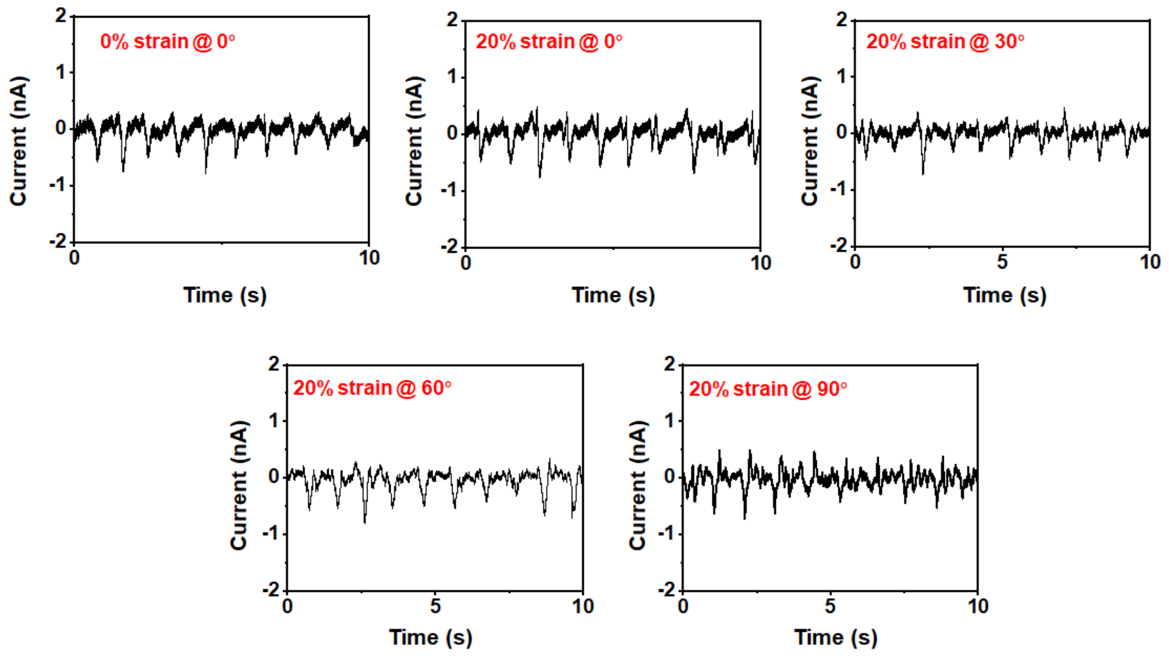

Figure S22: Current outputs of MF network PDMS films. Applying a gentle tapping force (3N) at 1 Hz, the current outputs were measured in MF network PDMS film without strain and with 20% strains by stretching from 0° to 90 °.

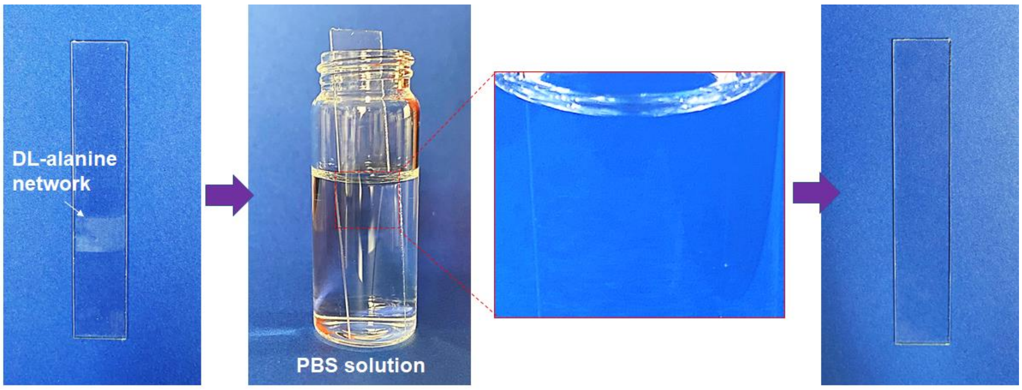

Figure S23: **DL-alanine MF Network Biodegradability.** Digital images of DL-alanine MF network before and after immersing into phosphate buffered saline (PBS) solution.

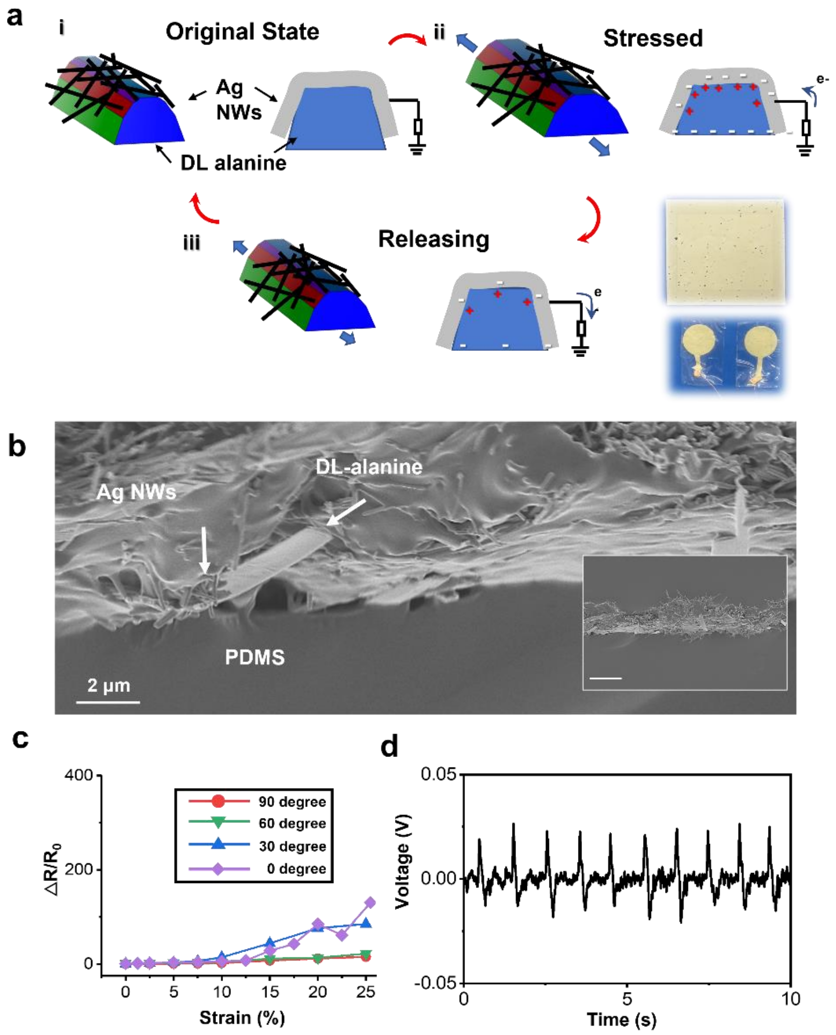

Figure S24: Fully Stretchable Soft Piezoelectric NG. a. Schematic of working mechanism of the single-electrode stretchable piezoelectric NG. i. Without stress, the charges between MF and Ag electrodes are balanced. ii. When stress is applied, the out-of-plane dipoles and accumulated positive charges on the lateral and top side of MFs draw negative charges from the ground into Ag NWs electrode. Iii. Once the stress is released, the induced dipole and surface charges disappears in MFs, which drive the negative charge on the Ag NWs to flow back to the ground. **b.** Cross-sectional SEM image of the piezoelectric NG. The DL-alanine MFs are seamlessly integrated with Ag NWs. Ag NWs have close contacts with DL-alanine micro-belt, suggesting the good ability to collect piezoelectric response. **c.** Electrical conductivity of the percolated Ag NWs at strains along all directions. This Ag NWs electrode can retain excellent conductivity with all directional strains up to 30%. **d.** Voltage output of the device per bending action at 1 Hz.

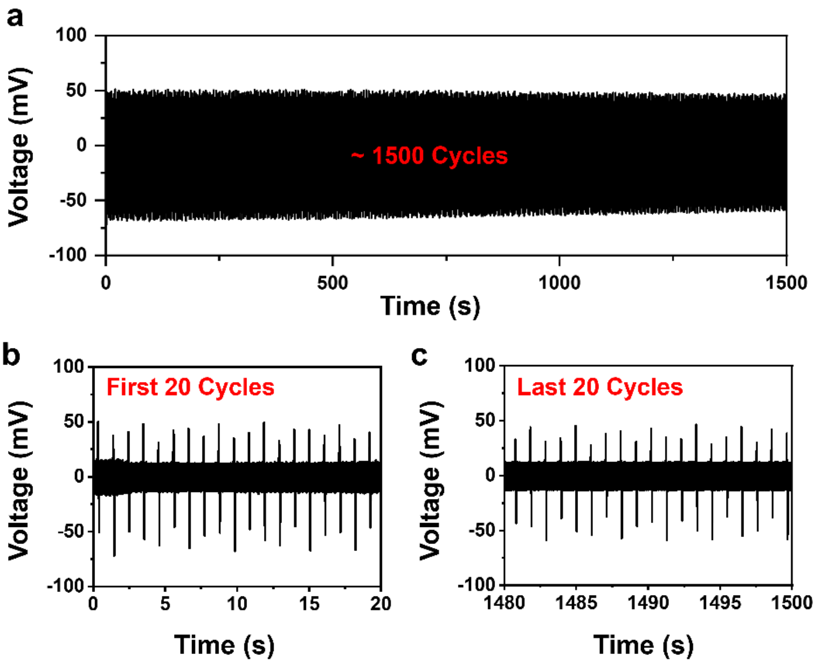

Figure S25. Long-term durability test of DL alanine network based piezoelectric device. a. Voltage outputs of 20% strained device under 1500 cyclic bending. **b.** Voltage outputs under first 20 cyclic bending. **c.** Voltage outputs under last 20 cyclic bending.

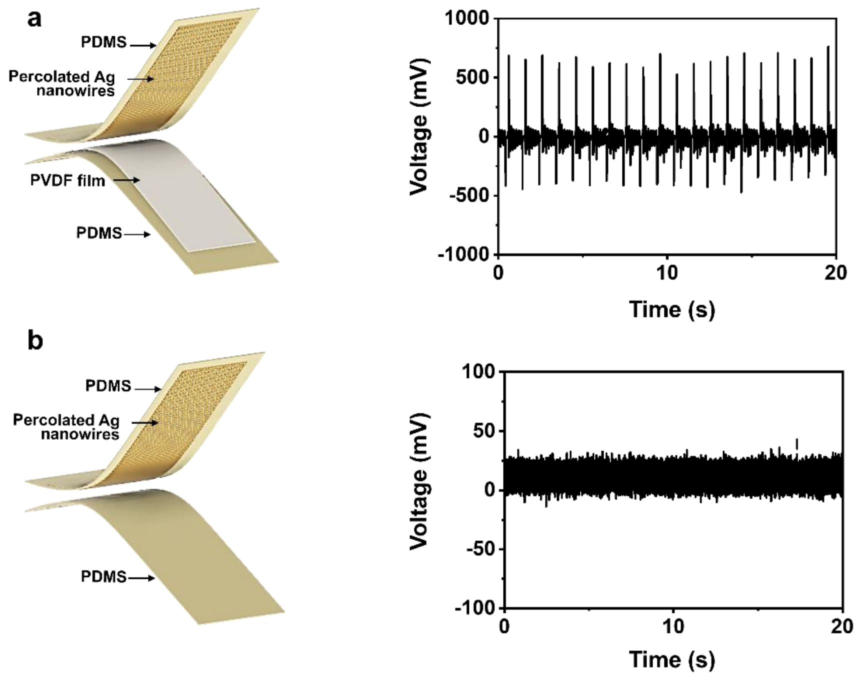

Figure S26. a. Schematic of PVDF based single electrode piezoelectric device and its output under knuckle bending. **b.** Schematic of bare PDMS based single electrode piezoelectric device and its output under knuckle bending.

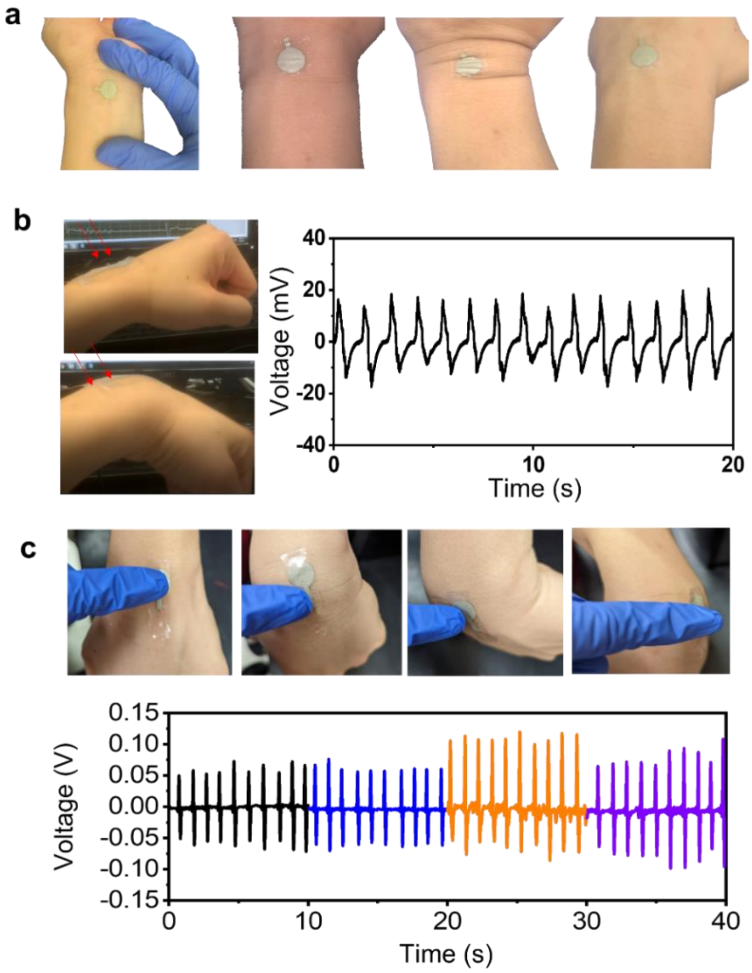

Figure S27: Stretchable Soft Piezoelectric NG Interfacing Skins. a. Digital image of DL-alanine piezoelectric NG placed on wrist. The device adhering to skin without delamination under different wrist movements. **b.** Digital images and voltage outputs of the device on wrist when the wrist was bended. **c.** Digital images and voltage outputs of the differently strained device on wrist when a gentle tapping by the finger is applied. Similar outputs of this device in response to finger touching are observed, suggesting the potential as artificial sensory receptors for body proprioception

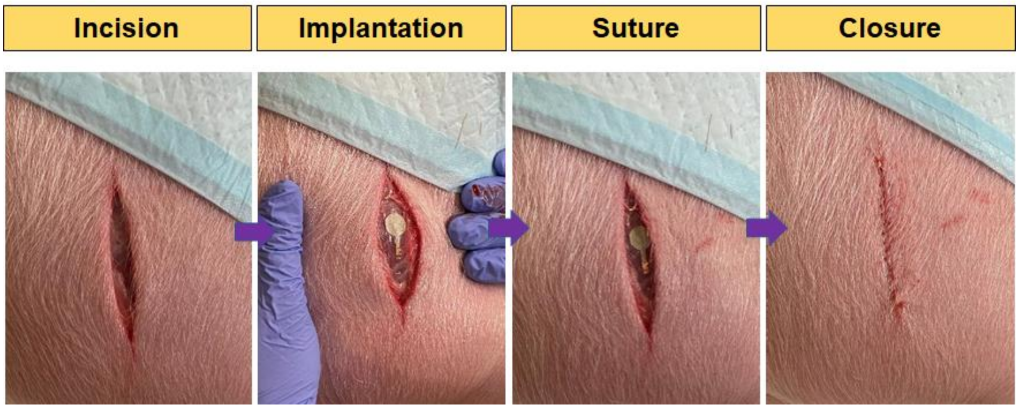

Figure S28: Surgical Processes of Piezoelectric NG Implantation in Swine Thigh.

Table S1. Comparison of Conformal Piezoelectric Device

Electromechanical Materials	Outputs /Force	Fabrication	Stretchability	Application	Efficiency	Ref
DL-alanine network	0.1 V/3 N	Self-assembly	20-40% all direction	In vivo sensor	1.08 %	this work
PZT kirigami	1 V/30 kPa	Template assisted sol-gel/poling	100% single direction	Joint sensor	-	8
P(VDF-TrFE)	0.7 V/-	Lithography/ spin-coating	30% single direction	Energy harvester	-	9
$BaTiO_3$-P(VDF-TrFE)	6 V/60 N	3D printing	300% single direction	Gait sensor	-	10
PZT ribbons	4 V/100 N	Lithographic patterning	Not stretchable	In vivo energy harvester	1.77%	41
ALN thin film	0.06 V/6 N	Lithographic patterning/Sputtering	Not stretchable	Facial Sensor	-	42
ALN (piezo)/PDMS (tribo)	8 V/5 N	Sputtering/UV patterning/Film assembly	120% single direction	Wearable Sensor	-	43
Boron nitride nanocomposite	5 V/40 N	3D printing/poling	Not stretchable	Robotic sensor	-	44

Supplementary References

1 Trzeciecki, M., Dähn, A. & Hübner, W. Symmetry analysis of second-harmonic generation at surfaces of antiferromagnets. *Physical Review B* **60**, 1144 (1999).

2 Maragkakis, G. M. *et al.* Nonlinear Optical Imaging of In-Plane Anisotropy in Two-Dimensional SnS. *Advanced Optical Materials* **10**, 2102776 (2022).
