# Supplementary material for: Stretchable piezoelectric biocrystal thin films
Source: Nat Commun. 2023 Oct 17;14:6562. doi: 10.1038/s41467-023-42184-8 (PMC10582159; doi:10.1038/s41467-023-42184-8)
Supplement: Supplementary file 5 — Reporting Summary [file 41467_2023_42184_MOESM5_ESM.pdf]

nature portfolio

Corresponding author(s): Xudong Wang

Last updated by author(s): 09-18-2023

Reporting Summary

Nature Portfolio wishes to improve the reproducibility of the work that we publish. This form provides structure for consistency and transparency in reporting. For further information on Nature Portfolio policies, see our Editorial Policies and the Editorial Policy Checklist.

Statistics

For all statistical analyses, confirm that the following items are present in the figure legend, table legend, main text, or Methods section.

n/a	Confirmed	
☐	☒	The exact sample size (*n*) for each experimental group/condition, given as a discrete number and unit of measurement
☐	☒	A statement on whether measurements were taken from distinct samples or whether the same sample was measured repeatedly
☒	☐	The statistical test(s) used AND whether they are one- or two-sided<br>*Only common tests should be described solely by name; describe more complex techniques in the Methods section.*
☒	☐	A description of all covariates tested
☒	☐	A description of any assumptions or corrections, such as tests of normality and adjustment for multiple comparisons
☐	☒	A full description of the statistical parameters including central tendency (e.g. means) or other basic estimates (e.g. regression coefficient) AND variation (e.g. standard deviation) or associated estimates of uncertainty (e.g. confidence intervals)
☒	☐	For null hypothesis testing, the test statistic (e.g. F, t, r) with confidence intervals, effect sizes, degrees of freedom and P value noted<br>*Give P values as exact values whenever suitable.*
☒	☐	For Bayesian analysis, information on the choice of priors and Markov chain Monte Carlo settings
☒	☐	For hierarchical and complex designs, identification of the appropriate level for tests and full reporting of outcomes
☒	☐	Estimates of effect sizes (e.g. Cohen's d, Pearson's r), indicating how they were calculated

Our web collection on statistics for biologists contains articles on many of the points above.

Software and code

Policy information about availability of computer code

Data collection	LabVIEW 2016, Keithley DMM 6500 multimeter, Stanford Research Systems low-noise current preamplifier, ANSYS 19.0 Version
Data analysis	Origin 2018 was used for data analysis and plotting.

For manuscripts utilizing custom algorithms or software that are central to the research but not yet described in published literature, software must be made available to editors and reviewers. We strongly encourage code deposition in a community repository (e.g. GitHub). See the Nature Portfolio guidelines for submitting code & software for further information.

Data

Policy information about availability of data

All manuscripts must include a data availability statement. This statement should provide the following information, where applicable:

- Accession codes, unique identifiers, or web links for publicly available datasets
- A description of any restrictions on data availability
- For clinical datasets or third party data, please ensure that the statement adheres to our policy

The authors declare that all data supporting the findings of this study are available within the Article and its Supplementary Information. The raw data generated in this study are available from the corresponding author upon reasonable request.

Research involving human participants, their data, or biological material

Policy information about studies with human participants or human data. See also policy information about sex, gender (identity/presentation), and sexual orientation and race, ethnicity and racism.

Reporting on sex and gender	Sex and gender are not considered in this study. This study only need to validate the functionality of wearable piezoelectric device on human object.
Reporting on race, ethnicity, or other socially relevant groupings	Race, ethnicity or other socially relevant groups are not considered in this study.
Population characteristics	Ages of population are between 20-30 years old.
Recruitment	Recruitment was conducted by interacting potential individuals, and screening. Although not critical, self-selection bias is minimized by randomization in this study as it would not affect the outputs of wearable piezoelectric device.
Ethics oversight	Approval for conducting human subjects research was obtained from the University of Wisconsin Institutional Review Board (IRB) prior to the start of this project, IRB protocol (ID: 2022-0805).

Note that full information on the approval of the study protocol must also be provided in the manuscript.

Field-specific reporting

Please select the one below that is the best fit for your research. If you are not sure, read the appropriate sections before making your selection.

☒ Life sciences ☐ Behavioural & social sciences ☐ Ecological, evolutionary & environmental sciences

For a reference copy of the document with all sections, see nature.com/documents/nr-reporting-summary-flat.pdf

Life sciences study design

All studies must disclose on these points even when the disclosure is negative.

Sample size	Three participants were recruited (n=3) for evaluating the wearability and performance of piezoelectric devices. The sample size of domestic pigs was three (n=3) for evaluating the performance of stretchable piezoelectric device in Fig 4. Two pigs were sufficient to demonstrate the capabilities of the device and collect all the data. Another pig was used for repeating the previously measured results. The sample size for each group in cell cytotoxicity study (Fig 3) was set as 6 (n=6). The size number was chosen to keep the chance of errors at an acceptably low level and a good reproducibility while avoiding making the study unnecessarily large.
Data exclusions	There are no data excluded from the analyses.
Replication	The replication of implantable piezoelectric device was successful. The stretchable piezoelectric device generated consistent outputs for repeated experiment. The replication of cytotoxicity was also successful. The DL alanine is not cytotoxic for all the repeated experiments.
Randomization	Randomization is not relevant to these experiment because the experimental goals are only to validate the functionality of this device and bio-compatibility of DL alanine materials. This is different from drug experiment in clinical trial where treatment group and control group included.
Blinding	Binding was not relevant to this study. Binding would not affect the performance of piezoelectric device.

Reporting for specific materials, systems and methods

We require information from authors about some types of materials, experimental systems and methods used in many studies. Here, indicate whether each material, system or method listed is relevant to your study. If you are not sure if a list item applies to your research, read the appropriate section before selecting a response.

Materials & experimental systems

n/a	Involved in the study
☒	☐ Antibodies
☐	☒ Eukaryotic cell lines
☒	☐ Palaeontology and archaeology
☐	☒ Animals and other organisms
☒	☐ Clinical data
☒	☐ Dual use research of concern
☒	☐ Plants

Methods

n/a	Involved in the study
☒	☐ ChIP-seq
☒	☐ Flow cytometry
☒	☐ MRI-based neuroimaging

Eukaryotic cell lines

Policy information about cell lines and Sex and Gender in Research

Cell line source(s)	Mouse vascular smooth muscle cells (MOVAS) were purchased from American Type Culture Collection (ATCC, CRL-2797)
Authentication	Cell cultures purchased from ATCC were authenticated by Short Tandem Repeat (STR) prior to purchase.
Mycoplasma contamination	Cell lines were not tested for mycoplasma contamination.
Commonly misidentified lines (See ICLAC register)	No commonly misidentified cell line in this work.

Animals and other research organisms

Policy information about studies involving animals; ARRIVE guidelines recommended for reporting animal research, and Sex and Gender in Research

Laboratory animals	Domestic pig. The species is Sus scrofa; the strain is Duroc, Landrace, Large white. The age of swine is 3 months old.
Wild animals	The study did not involve wild animals.
Reporting on sex	Sex was not considered in study design.
Field-collected samples	The study did not involve samples collected from the field.
Ethics oversight	All animal experiments were conducted under a protocol approved by the University of Wisconsin Institutional Animal Care and Use Committee.

Note that full information on the approval of the study protocol must also be provided in the manuscript.
