# Supplementary material for: Stretchable piezoelectric biocrystal thin films
Source: Nat Commun. 2023 Oct 17;14:6562. doi: 10.1038/s41467-023-42184-8 (PMC10582159; doi:10.1038/s41467-023-42184-8)
Supplement: Supplementary file 6 — Description of Additional Supplementary Files Document [file 41467_2023_42184_MOESM6_ESM.pdf]

<u>Description of Additional Supplementary Files</u>

Supplementary Movie 1 to 3

Supplementary Movie 1: Large-scale microstructure displacement when stretching a DL-alanine MF network

Supplementary Movie 2: Zoomed-in microstructure displacement when stretching a DL-alanine MF network

Supplementary Movie 3: A stretchable piezoelectric NG on knuckle in response to different bending
